# P-170. Increasing dengue virus seroprevalence in a cohort of agricultural workers in rural southwest Guatemala

**DOI:** 10.1093/ofid/ofaf695.394

**Published:** 2026-01-11

**Authors:** Blair Weikel, Julio del Cid-Villatoro, Neudy C Rojop, Daniel Vásquez, Cassandra Waltman, Claire Bradley, Chandler Bradley, Mattie Cassaday, Prem Lakshmanane, Rosemary Rochford, May Chu, Ross Kedl, Molly Lamb, Daniel Olson

**Affiliations:** University of Colorado Anschutz Medical Campus, Aurora, CO; Fundación para la Salud Integral de los Guatemaltecos, Retalhuleu, Retalhuleu, Guatemala; Fundacion Para La Salud Integral de los Guatemaltecos, Los Encuentros, Retalhuleu, Guatemala; Fundación para la Salud Integral de los Guatemaltecos, Retalhuleu, Retalhuleu, Guatemala; University of Colorado Anschutz, Aurora, Colorado; Fundación para la Salud Integral de los Guatemaltecos, Retalhuleu, Retalhuleu, Guatemala; University of Colorado School of Medicine, Aurora, Colorado; University of Colorado Department of Immunology & Microbiology, Aurora, Colorado; University of North Carolina, Chapel Hill, North Carolina; University of Colorado, Anschutz Medical Campus, Aurora, Colorado; University of Colorado Anschutz Medical Campus, Aurora, CO; University of Colorado Department of Immunology & Microbiology, Aurora, Colorado; Colorado School of Public Health, Aurora, Colorado; CU School of Medicine, Denver, Colorado

## Abstract

**Background:**

In 2024, Guatemala declared a national public health emergency for dengue virus (DENV) in response to a 5-fold increase in cases. As most infections are asymptomatic, it is difficult to estimate the true underlying seroprevalence of infection. Knowing individual- and population-level serostatus is critical for vaccine intervention efforts, as only those who are seropositive should receive the vaccine. Understanding characteristics of the seropositive population will help target disease-prevention interventions.

Overall Seroprevalence of Primary DENV Infection by Year

**Figure:**
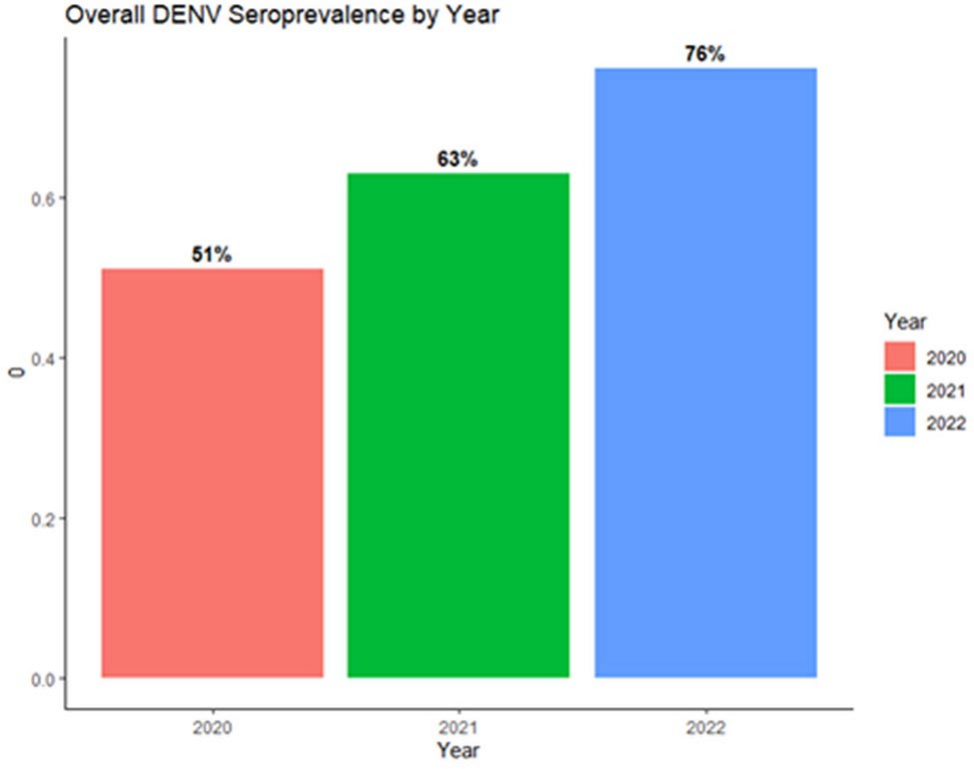

**Figure:**
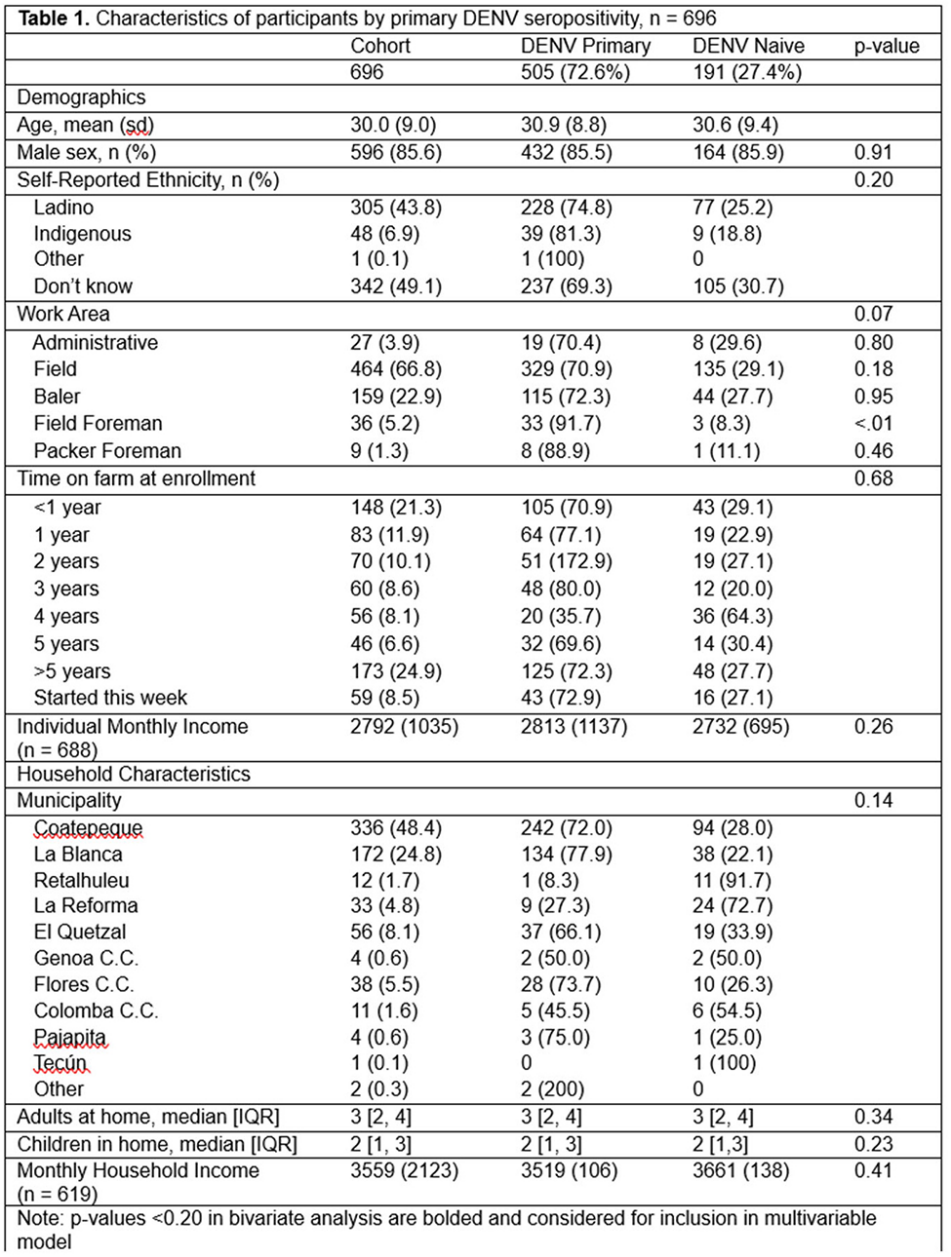

**Methods:**

We analyzed serum blood samples from the AGRI cohort, a longitudinal study of agricultural workers in southwest Guatemala, using an IgG-based multiplex bead assay conjugated to DENV1-4/ZIKV EDIII. A decision-tree algorithm classified infection as primary DENV if the mean fluorescence intensity (MFI) for a DENV serotype accounted for >33% of total MFI for all flaviviruses. We calculated annual population-level seroprevalence of primary DENV infection, adjusting for test sensitivity and specificity, and examined associations between seropositivity and demographic, occupational, and socioeconomic factors.

**Figure:**
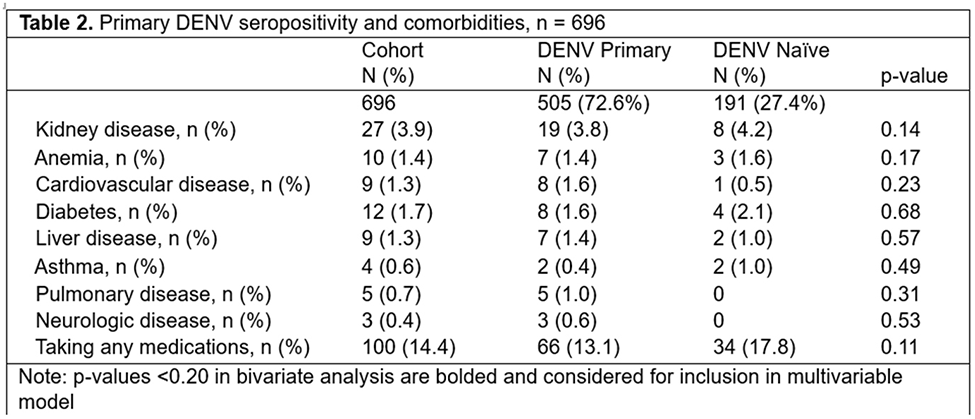

**Results:**

Among 696 participants with at least one sample, primary DENV seroprevalence increased from 51% in 2020 to 63% in 2021 and 76% in 2022. There was a significant difference in primary DENV seropositivity among field foremen (92% seropositive, 8% seronegative, p< 0.01) vs other farm job types (approximately 70% seropositivity). No other significant associations were found.

**Conclusion:**

We currently have an additional ∼7,000 samples collected through 2024 in laboratory analysis and will be incorporating geospatial data for spatial analysis. While specific risk factors remain unclear, our findings highlight increasing DENV seroprevalence in the population, underscoring the need for ongoing surveillance and targeted interventions.

**Disclosures:**

Blair Weikel, MPH, Merck: Grant/Research Support Molly Lamb, PhD, Merck: Grant/Research Support Daniel Olson, MD, Fundacion para la Salud Integral de los Guatemaltecos: Board Member|Merck: Grant/Research Support|Roche Diagnostics: Grant/Research Support

